# Clinical Features and Dynamics of T Cells-Related Markers in Immunocompetent Patients with Cytomegalovirus Hepatitis

**DOI:** 10.1155/2020/8874620

**Published:** 2020-08-30

**Authors:** Takuya Komura, Takashi Kagaya, Hideo Takayama, Masahiro Yanagi, Takatoshi Yoshio, Saiho Sugimoto, Michiko Nishino, Noriaki Orita, Yoshiro Asahina, Masashi Nishikawa, Shuichi Kaneko, Masashi Unoura

**Affiliations:** ^1^Division of Gastroenterology NHO, Kanazawa Medical Center, Kanazawa, Japan; ^2^System Biology, Kanazawa University, Graduate School of Medical Science, Kanazawa, Japan

## Abstract

**Aim:**

Cytomegalovirus (CMV) can cause hepatitis, encephalomyelitis, and pneumonitis in immunocompromised patients. In contrast, CMV infection of immunocompetent patients can lead to the development of infectious mononucleosis and is typically self-limiting; severe complications are rare. We evaluated the pathophysiology and immunological aspects of CMV hepatitis in recently immunocompetent adult patients.

**Methods:**

We examined the clinical features and outcomes of 47 adult immunocompetent patients with CMV hepatitis (29 men, 18 women; mean age, 34 ± 11 years) from January 2005 to August 2019 treated in our hospital. We also assayed T-cell activation to evaluate the immune responses in these patients.

**Results:**

Fever (74.5%), hepatosplenomegaly (74.5%), sore throat (36.2%), headache (31.9%), abdominal pain (27.7%), lymphadenopathy (23.4%), and skin rash (6.4%) were present at admission. Complications included gastrointestinal injury (25.5%), neuropathy (4.3%), thrombocytopenia (2.1%), and splenic infarction (2.1%). All patients had a good clinical course without liver failure or transition to chronic liver injury. The time to recover from liver injury ranged from 12 to 142 days (mean, 43.4 ± 28.7 days). The serum sIL-2R level, which reflects T-cell activation, was transiently elevated and correlated with the extent of hepatic inflammation.

**Conclusions:**

CMV hepatitis in immunocompetent individuals has a satisfactory outcome, but occasionally results in complications in other organs. The sIL-2R level has potential as a surrogate marker of hepatic inflammation in immunocompetent patients with CMV hepatitis.

## 1. Introduction

Reactivation of latent cytomegalovirus (CMV) infection or acquisition of primary CMV infection can result in encephalitis, pneumonitis, hepatitis, uveitis, retinitis, colitis, and graft rejection in immunocompromised patients [1]. In contrast, in immunocompetent patients, primary CMV infection typically manifests as an undifferentiated viral syndrome or an infectious mononucleosis-like syndrome. Therefore, CMV infection in such individuals is considered to be of minimal importance [2].

The seroprevalence of CMV in immunocompetent young adults worldwide has decreased to approximately 60% from 70–100% [3]. Therefore, the rate of primary CMV infection has increased recently. The disease is considered to be benign and self-limiting in immunocompetent individuals; however, some recent reports have indicated that CMV infection of immunocompetent patients can result in severe clinical manifestations [4]. Thus, further research is needed regarding CMV infection in recently immunocompetent individuals.

However, CMV infection induces large numbers of CD8^+^ T cells that retain effector functions and are dependent on CD4^+^ T cells help and costimulatory signals that home to peripheral organs [5]. The level of the soluble interleukin-2 receptor (sIL-2R) in peripheral blood reflects the extent of T-cell activation [6], which is correlated with the development of malignant lymphoma [7], rheumatoid arthritis [8], and IgG4-related disease [9]. However, the immune response in immunocompetent patients with CMV hepatitis is unclear.

In the present study, we examined the clinical features and outcomes of CMV-infected immunocompetent patients and assessed the immunological response to CMV infection by assaying the sIL-2R level and CD4/CD8 T-cells ratio.

## 2. Materials and Methods

The study was performed in accordance with the ethical guidelines of the 2013 Declaration of Helsinki; the study protocol was approved by the Institutional Review Board of the NHO Kanazawa Medical Center.

### 2.1. Patients

This study included 47 immunocompetent patients with CMV hepatitis (29 men, 18 women; mean age, 34 ± 11 years) who were diagnosed and treated at NHO Kanazawa Medical Center from January 2005 to August 2019 (Table 1). Immunocompetence was defined as the absence of current or prior malignant disease, steroid or immunomodulatory treatment, or immune disorders. All patients were positive for anti-CMV IgM in serum or for CMV antigenemia. Patients with liver injuries due to other etiologies were excluded. The serum sIL-2R level was assessed in 24 patients with CMV infection (17 men, 7 women; mean age, 61 ± 7 years) (Table 2). There were no differences in any measured parameter between males and females.

### 2.2. Clinical Features

At admission, we evaluated the following factors: white blood cell (WBC) count, frequency of lymphocytes, ratio of atypical lymphocytes, maximum C-reactive protein (CRP) level, maximum aspartate aminotransferase level (AST), maximum alanine aminotransferase (ALT) level, maximum total bilirubin (T-bil) level, minimum albumin (Alb) level, maximum prothrombin time- (PT-) international normalized ratio (INR), injuries to other organs, treatments, transition to hepatic failure, transition to chronic liver injury, interval to amelioration of hepatitis, and clinical outcome. Amelioration of hepatitis was defined as normalization of the serum ALT level.

### 2.3. Immunological Parameters

#### 2.3.1. T-Cell Activation

In patients with CMV infection, CD8^+^ T-cells responses are dependent on CD4^+^ T-cells aid and costimulatory signals. We assayed serum sIL-2R levels using the STACIA CLEIA kit (LSI Medience Corporation, Tokyo, Japan; normal range: 121–613 U/mL) in 24 patients at admission and its correlations with the following factors: WBC count, lymphocyte count, CRP level, AST level, ALT level, T-bil level, minimum Alb level, and maximum PT-INR level. Finally, we examined the serum sIL-2R level after amelioration of hepatitis.

#### 2.3.2. Other Markers of Infection

We assayed the serum human immunodeficiency virus (HIV) antibody level in 15 patients. We also evaluated the frequencies of CD4^+^ and CD8^+^ T cells at admission and after amelioration of hepatitis in six patients. Six patients who provided written informed consent for evaluation of their CD4^+^ and CD8^+^ T-cells numbers were enrolled. We measured the plasma HIV RNA level using the Roche TaqMan assay (lower limit of detection, 20 copies/mL); we assayed CD4^+^ and CD8^+^ T-cells subsets by flow cytometry. Serum sIL-2R levels and CD4^+^ and CD8^+^ T-cell counts are routinely measured in our hospital when needed. These parameters served as measures of T-cell activation.

### 2.4. Statistical Analysis

Statistical analysis was performed using Microsoft Excel software (Microsoft, Redmond, WA, USA). Data are shown as medians and interquartile ranges, as well as means and standard errors of the mean. Differences in variables before and after normalization of the ALT level were examined by paired Student's *t*-tests after a normal distribution had been confirmed. A value of *P* < 0.05 was considered indicative of statistical significance. The Pearson correlation coefficient was calculated to assess correlations of the serum sIL-2R level with a variety of laboratory parameters.

## 3. Results

### 3.1. Demographics

**Figure 1:**
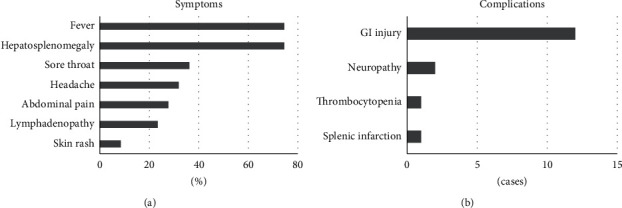
Some clinical symptoms and complications of CMV hepatitis in 47 immunocompetent individuals. (a) The frequency of some clinical symptoms is shown. Fever, 35 patients (74.5%); hepatosplenomegaly, 35 patients (74.5%); sore throat, 17 patients (36.2%); headache, 15 patients (31.9%); abdominal pain, 13 patients (27.7%); lymphadenopathy, 11 patients (23.4%); and skin rash, 4 patients (6.4%) on arrival to our hospital, respectively. (b) The frequency of some clinical complications is shown. GI injury, 7 patients (6.4%); neuropathy, 2 patients (6.4%); thrombocytopenia, 1 patient (6.4%); and splenic infarction, 1 patient (6.4%), respectively.

We retroactively assessed 47 immunocompetent patients with CMV hepatitis. The demographic characteristics of the patients are shown in Table 1. The serum AST and ALT levels were moderately elevated, and the CRP level was slightly elevated. The T-bil, Alb, and PT-INR levels were not decreased, suggesting that the hepatic reserve was not impaired. Fever, hepatosplenomegaly, sore throat, headache, abdominal pain, lymphadenopathy, and skin rash at admission were found in 35 (74.5%), 35 (74.5%), 17 (36.2%), 15 (31.9%), 13 (27.7%), 11 (23.4%), and 4 (6.4%) patients, respectively (Figure 1(a)). Gastrointestinal (GI) injury occurred in 12 patients (25.5%), neuropathy in two patients (4.3%), thrombocytopenia in one patient (2.1%), and splenic infarction in one patient (2.1%) (Figure 1(b)).

### 3.2. Outcomes

All patients survived; one was treated with ganciclovir (an anti-CMV agent) for 10 days because of a high fever and severe general malaise caused by bacteremia. No patient developed hepatic failure or chronic liver injury. In addition, no patients showed a worsened serum PT-INR or Alb level. The serum T-bil levels in two patients were 3.5 and 3.2 mg/dL, and thus slightly elevated. The ALT levels of all patients normalized after 43.4 ± 28.7 days from the peak.

We observed several complications associated with CMV infection in the absence of hepatitis. Gastrointestinal (GI) injury was classified as gastric ulcer and erosion (seven patients), duodenal ulcer and erosion (two patients), esophageal ulcer (one patient), and colitis (two patients); the diagnoses were pathologically proven by endoscopically obtained biopsy. Antacids were given to 10 patients. The other symptoms were treated palliatively. Two patients became neurological diseases. One patient was diagnosed with Guillain–Barre syndrome and was prescribed an oral steroid and *γ*-globulin for 5 days. Another patient exhibited mild neuropathy, but the clinical course was good in the absence of treatment. One patient developed thrombocytopenia, and 10 units of platelet concentrate were infused; the clinical course was good. The patient with splenic infarction did not require any treatment and exhibited a good clinical course.

### 3.3. Immunological Parameters

**Figure 2:**
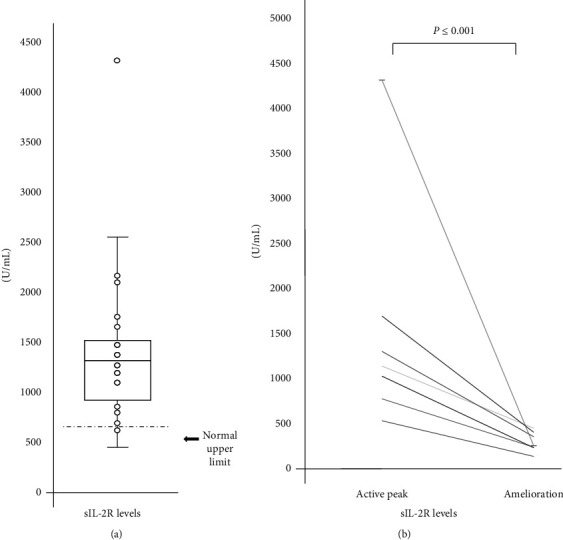
sIL-2R levels in serum of 24 patients with CMV hepatitis. (a) sIL-2R levels in serum of patients with CMV hepatitis were elevated on arrival. Dot indicated each sIL-2R levels of CMV patients. Data are expressed as means ± SEM. (b) 7 patients could be measured at sIL-2R levels after normalization of ALT levels which suggested calm down of hepatitis. These levels were significantly decreased. ^*∗*^*P* < 0.05.

**Figure 3:**
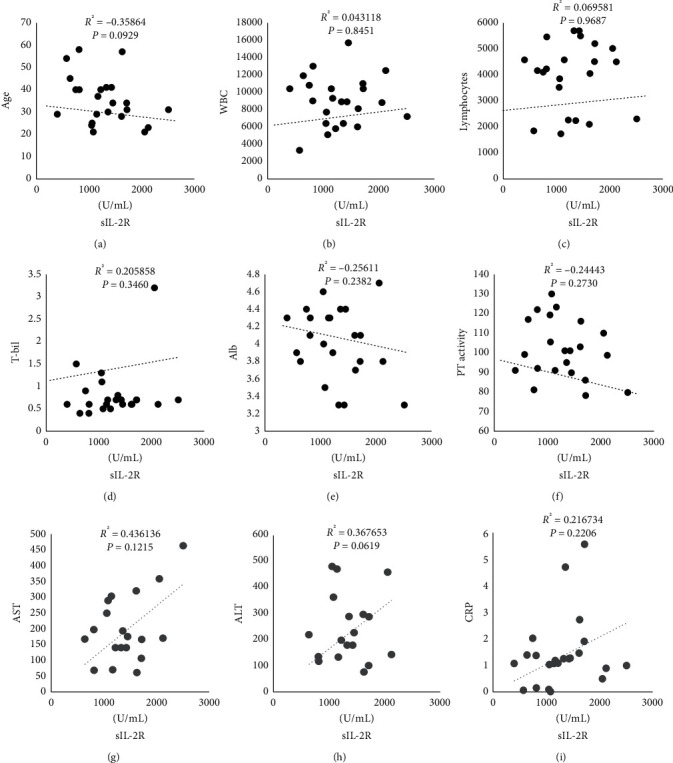
Correlation with sIL-2R levels and some laboratory data in peripheral blood of 24 patients with CMV hepatitis. (a) Age (years), (b) WBC counts (/mm^3^), (c) lymphocytes counts (/mm^3^), (d) T-bil (mg/dL), (e) Alb (g/dL), (f) PT activity (%), (g) AST (IU/L), (h) ALT (IU/L), and (i) CRP (mg/dL), respectively. (d–f) Hepatitis reserve ability.

The characteristics of the study patients are shown in Table 2. The serum sIL-2R level was significantly elevated at the peak of hepatitis (range 399–4,287 U/mL; mean 1,414 U/mL; Figure 2(a)) but became normal after amelioration of hepatitis (Figure 2(b)). The sIL-2R level was correlated moderately with the AST, ALT, and CRP levels, which reflect liver inflammation and damage (Figure 3(a)), and was correlated weakly with the serum T-bil, Alb, and PT-INR levels (Figure 3(b)), which reflect the liver reserve. The serum sIL-2R level, which reflects T-cell activation, was transiently elevated and correlated with disease activity.

**Figure 4:**
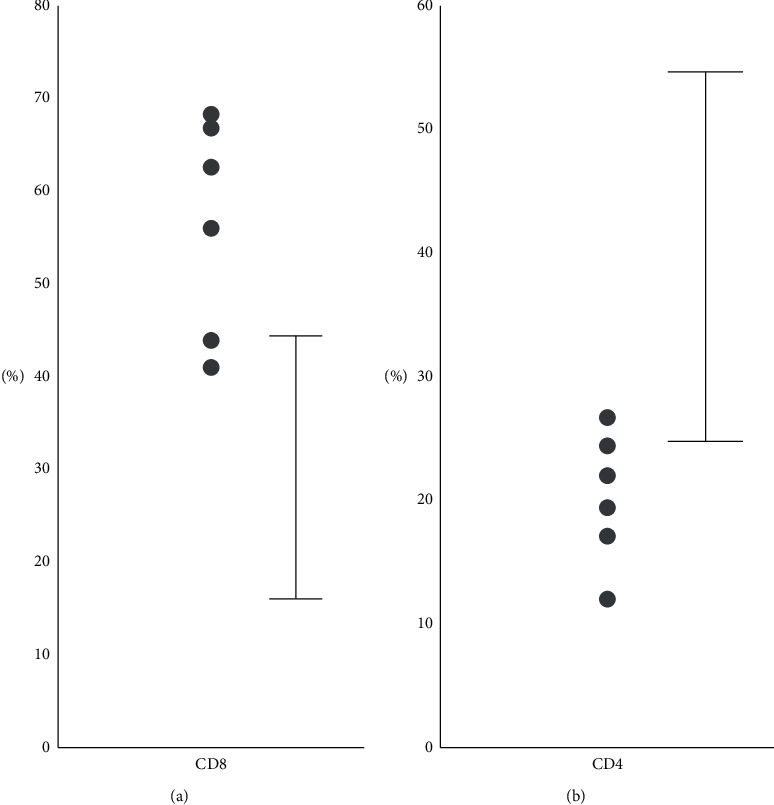
The CD4/CD8 ratio in the peripheral blood of six patients with CMV hepatitis, measured at the time of admission. (a) The CD8^+^ T-cell proportions were high. (b) The CD4^+^ T-cell proportions were low. The solid lines indicate the normal ranges of the CD4^+^ and CD8^+^ T-cell proportions.

No anti-HIV antibodies were detected. The frequency of CD8^+^ T cells was transiently elevated (Figure 4(a)), while the frequency of CD4^+^ T cells was reduced, during active hepatitis (Figure 4(b)). Following amelioration of hepatitis, the (CD4/CD8) ratio became normalized (data not shown).

## 4. Discussion

All immunocompetent patients with CMV hepatitis exhibited transient symptoms, although antivirals were typically not required. While most patients exhibited a benign clinical course, some developed complications that required palliative treatment.

It is often considered that CMV infection in immunocompetent individuals is self-limiting and does not require treatment [10]. However, we detected a variety of common complications of CMV infection, including GI injury, neuropathy, and thrombosis [11–13]. The GI injuries were mild and were treated successfully. Vascular thrombosis is a potential manifestation of CMV infection in immunocompetent individuals. A variety of veins can be affected by CMV infection, presumably due to the procoagulant activity of CMV [14, 15]. The suppression of hematopoiesis associated with CMV infection is caused by direct inhibition of the growth of progenitor hematopoietic cells, resulting in immunological abnormalities and thrombocytopenia [16]. A potential reason for the relatively large number of complications observed in our study was that we chose patients with CMV-induced hepatitis, all of whom exhibited CMV infection-induced organ injury; thus, they likely had severe CMV infection. Hence, CMV infection in immunocompetent patients can occasionally trigger relatively severe complications and should be treated aggressively [11].

IL-2 is a multipotent cytokine that plays a crucial role in signaling pathways that control the differentiation and homeostasis of pro- and anti-inflammatory T cells by binding with high affinity to trimeric IL-2R. Hence, sIL-2R reflects the level of T-cell activation [17]. The sIL-2R level was correlated with the AST, ALT, CRP, T-bil, Alb, and PT-INR levels, suggesting that the sIL-2R level reflects the extent of CMV hepatitis activity in infected patients, as in those with systemic lupus erythematosus and sarcoidosis, both of which feature T-cell activation [18, 19]. The transient decrease in the (CD4/CD8) ratio is in line with that of an earlier report suggesting that CMV infection increases the numbers of CD8^+^ T cells that exert effector functions [5].

In conclusion, patients with CMV hepatitis had satisfactory outcomes, such that few required antiviral agents. However, some complications required transient palliative treatment. The serum sIL-2R level, which reflects the T-cell response, was identified as a biomarker of disease activity in immunocompetent patients with CMV hepatitis.

## Figures and Tables

**Table 1:** Characteristics of the study subjects.

Characteristics	
Gender, male/female	29/18
Age	34.0 ± 10.9 (20–58)
White blood cell counts	9800 ± 4000/mm^3^
Lymphocyte ratio	43.0 ± 12.0%
Atypical lymphocyte ratio	28 (53.6%)
Maximum AST	239 ± 172 IU/L
Maximum ALT	340 ± 231 IU/L
Maximum CRP	1.5 ± 1.6 mg/dL
Maximum T-bil	0.84 ± 0.66 mg/dL
Minimal Alb	4.1 ± 0.40 g/dL
Maximum PT-INR	1.00 ± 0.094

Data are expressed as means ± SD.

**Table 2:** Characteristics of the sIL-2R study subjects.

Characteristics	
Gender, male/female	17/7
Age	35.3 ± 10.3 (21–58)
White blood cell counts	9033 ± 2751/mm^3^
Lymphocyte ratio	45.8 ± 10.4%
Atypical lymphocyte ratio	7.7 (31%)
Maximum AST	270 ± 177 IU/L
Maximum ALT	364 ± 258 IU/L
Maximum CRP	1.5 ± 1.3 mg/dL
Maximum T-bil	0.94 ± 0.77 mg/dL
Minimal Alb	4.0 ± 0.4 g/dL
Maximum PT-INR	0.98 ± 0.097

Data are expressed as means ± SD.

## Data Availability

The data used to support the findings of this study are included within the article.
